# Mental Health Self-Stigma of Syrian Refugees With Posttraumatic Stress Symptoms: Investigating Sociodemographic and Psychopathological Correlates

**DOI:** 10.3389/fpsyt.2021.642618

**Published:** 2021-07-13

**Authors:** Jonathan Bär, Alexander Pabst, Susanne Röhr, Melanie Luppa, Anna Renner, Michaela Nagl, Judith Dams, Thomas Grochtdreis, Anette Kersting, Hans-Helmut König, Steffi G. Riedel-Heller

**Affiliations:** ^1^Institute of Social Medicine, Occupational Health and Public Health, University of Leipzig (ISAP), Leipzig, Germany; ^2^Global Brain Health Institute, Trinity College Dublin, Dublin, Ireland; ^3^Department of Psychosomatic Medicine and Psychotherapy, University Medical Center Leipzig, Leipzig, Germany; ^4^Department of Health Economics and Health Services Research, Hamburg Center for Health Economics, University Medical Center Hamburg-Eppendorf, Hamburg, Germany

**Keywords:** self-stigma, mental health, Syrian refugees, posttraumatic stress, comorbidity

## Abstract

**Background:** The high prevalence of mental disorders related to posttraumatic stress among Syrian refugees is often in contrast with their low utilization of mental health care in the host countries. Mental health self-stigma, i.e., internalized stigma of having a mental disorder, could prevent individuals from seeking mental health care. Therefore, we aimed to provide evidence on different aspects of mental health self-stigmatization among adult Syrian refugees with posttraumatic stress symptoms residing in Germany. Moreover, we investigated associations with sociodemographic and psychopathological variables in order to identify those at higher risk of self-stigmatization.

**Material and Methods:** Overall, 133 participants with mild to moderate posttraumatic stress symptoms were recruited in the metropolitan areas of Leipzig, Dresden and Halle, Germany, using a multimodal approach. Mental health self-stigma was assessed using the Self-Stigma of Mental Illness Scale – Short Form (SSMIS-SF), consisting of four subscales (*Stereotype awareness, Stereotype agreement, Application to self* , *Harm to self-esteem*), each scoring from 5 (low) to 45 (high) points. Linear regression analysis was used to test associations of sociodemographic and psychopathological variables with self-stigma subscales.

**Results:** On average, self-stigma ratings ranged from 16.5 (SD = 6.6) points on *Application to self* to 28.3 (SD = 7.5) points on *Stereotype awareness*. Results showed higher scores on *Application to self* for individuals who were younger (*t* = 2.65, *p* = 0.009) and single (*F* = 5.70, *p* = 0.004). Regression analyses yielded statistically significant associations between having multiple comorbidities and a higher *Application to self* stigma (β = 0.18, *p* = 0.044), controlling for sociodemographic covariates.

**Discussion:** Mental health self-stigma was increased among Syrian refugees in Germany. Correlates of increased self-stigma could inform efforts to improve access to mental health care among Syrian refugees with mental ill-health. Longitudinal studies following an intersectional approach by concurrently examining multiple forms of public and internalized stigma could provide helpful insights for developing tailored stigma reduction efforts in this context.

## Introduction

Around the globe, the number of individuals forcibly displaced from their homes due to persecution, violence, conflict and human rights violations has risen to 79.5 million by the end of 2019 (1). Germany has been a major host for refugees for many years now, especially for individuals from Syria (2). Since the beginning of the civil war in Syria in 2011, around 790,000 refugees have sought protection in Germany (3). Upon arrival in a host country, refugees can be confronted with a multitude of post-migration stressors, such as insecure residential status, language barriers or the loss of social contacts (4). As a consequence of the distressing experiences before, during and after the escape from the origin country, a substantial proportion of individuals seeking asylum often experience posttraumatic stress and related mental ill-health. A study on mental health in Syrian refugees residing in Germany found that more than 30% met the criteria for at least one mental disorder (5). In contrast to the corresponding treatment needs, a recent systematic review showed a strong underutilization of mental health care in refugees and asylum seekers in European countries (6). Besides structural barriers in accessing health care (e.g., unstable living conditions, financial strain, language barriers), stigma related to mental health is considered to be a major barrier to help-seeking in refugee populations (7).

Sociologist Erving Goffman defined stigma as an “attribute that is deeply discrediting,” resulting in the “situation of the individual who is disqualified from full social acceptance” (8). Departing from Goffman's introduction of the stigma concept to the social sciences, different definitions and dimensions of stigma and the process of stigmatization have been discussed (9). In the context of stigma related to mental health, often denoted as mental illness stigma or mental health stigma, two fundamental dimensions of stigma have been differentiated: public stigma and self-stigma (10). Public stigma represents the stereotypes, prejudice and discrimination that society places on the stigmatized group. Self-stigma, in turn, denotes the internalization of the public stigma among individuals belonging to the stigmatized group. Corrigan and colleagues (11, 12) have described self-stigmatization as a process following four consecutive and interrelated stages: (I) awareness of stereotypes existing in the general public (i.e., perceived public stigma), (II) agreement to those stereotypes, (III) applying the stereotypes to oneself, and (IV) the consequent experience of harm as a decrement of self-esteem. Two central assumptions of this progressive model of self-stigma are: the trickle-down nature of the self-stigmatization process (with lower scores for each subsequent stage) and larger correlations between more proximal than between more distal stages. Testing these assumptions in different diagnostic groups (e.g., patients with serious mental illness, patients with addictive disorders) yielded mixed evidence, from confirming both assumptions to differences between “earlier” and “later” stages, but not between each of the four individual stages (12–15).

**Figure 1:**
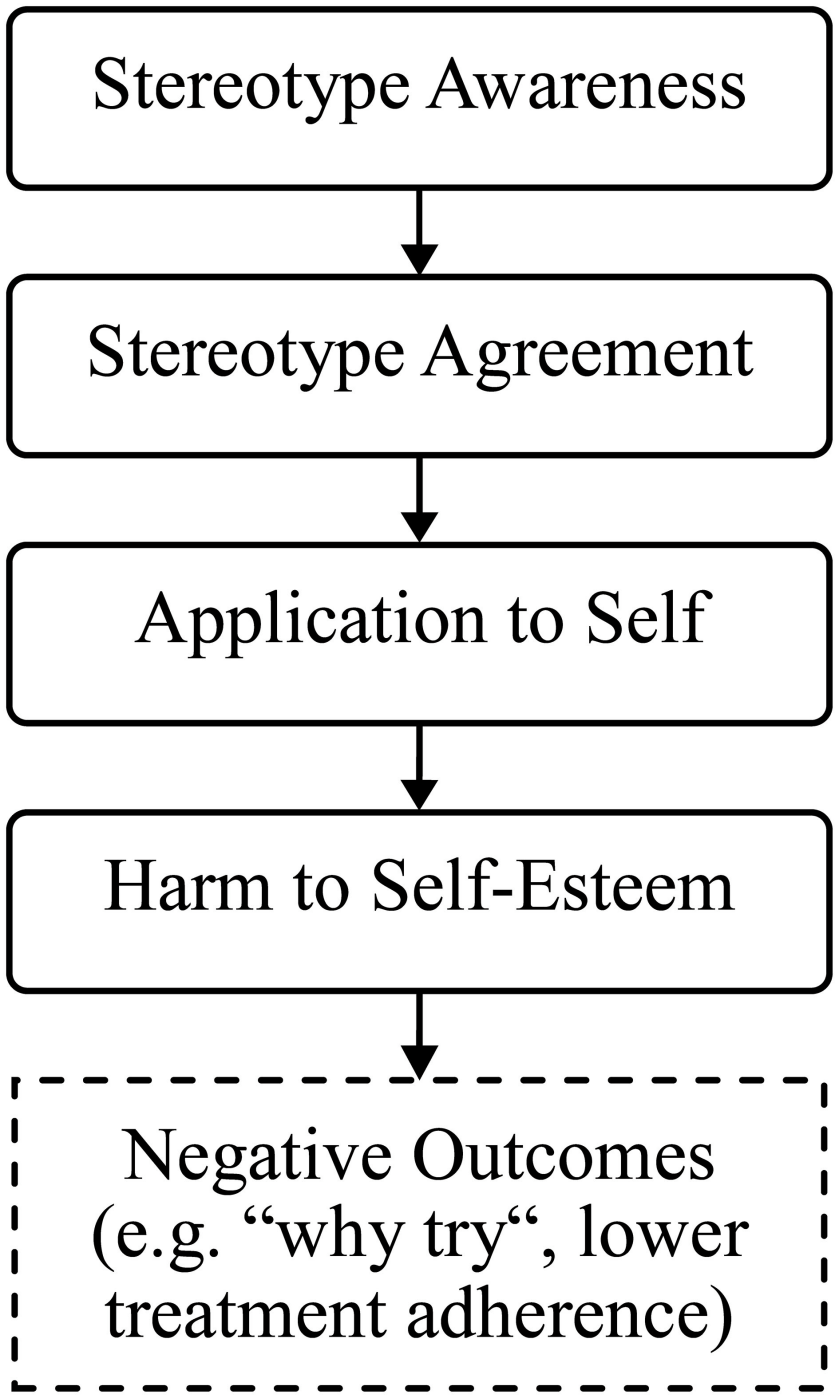
Progressive model of self-stigma (adapted from Corrigan et al. (16)).

The process of internalizing negative views among individuals with mental ill-health can have detrimental psychosocial effects (see Figure 1). For example, mental health self-stigmatization has been found to be associated with more severe psychopathological symptoms and lower treatment adherence (17), a sense of futility or “why try” effect (16) and suicidal ideation (18). Refugees with mental ill-health could be particularly vulnerable in this respect as they might experience intersecting stigmata, both as mentally ill and as potentially marginalized group in the host country (19). Despite the apparent gap between mental health care needs and actual utilization, the self-stigma of refugees experiencing posttraumatic stress is poorly understood. Research on self-stigma of individuals with posttraumatic stress symptoms has largely focused on veteran samples (20), with limited comparability to traumatic experiences and associated self-stigmatizing beliefs of specific refugee groups. Culture-dependent variations in correlates of mental health self-stigma have been previously reported (21), yet relations between cultural factors and mental health self-stigma remain unclear (22). In this regard, researchers have also pointed out an underrepresentation of individuals from Arabic countries in psychological research (23).

As for Syrian individuals with mental ill-health, the few existing studies suggest a high level of stigma related to undergoing specialized mental health treatments and receiving a mental disorder diagnosis. For example, Hassan and colleagues (24) have pointed out that being labeled as mentally ill in Syria bears the risk of being viewed as “mad” or “crazy,” which tends to be associated with concerns about bringing shame to oneself and one's family. Similar attitudes of the general public in Syria were reported in a recent qualitative study based on focus groups of Syrian refugees residing in Germany (25). The findings also confirmed strongly stigmatizing views about seeking help from mental health professionals. To date, it is an unresolved question how these stigmatizing beliefs in the general public in Syria translate to self-stigmatization of Syrian refugees with posttraumatic stress symptoms residing in Germany. To the best of our knowledge, no previous study has examined the applicability of the progressive model of self-stigma (11, 12) neither for individuals with posttraumatic stress symptoms nor refugee samples. In light of this lack of evidence, further improving our understanding of characteristics and correlates of mental health self-stigmatization within this group seems imperative.

Against this background, we aimed to provide an explorative analysis of mental health self-stigma among adult Syrian refugees with mild to moderate posttraumatic stress symptoms residing in Germany, drawing on cross-sectional baseline data from the *Sanadak* trial (26). The *Sanadak* trial comprised the development and evaluation of a low-threshold Arabic-language self-help app for posttraumatic stress, targeting Syrian refugees residing in Germany. Our objectives were: (I) to describe the level of mental health self-stigma among Syrian refugees according to the progressive model of self-stigma, (II) to investigate possible differences in mental health self-stigma according to age, gender, education and family status, and (III) to determine whether individuals were more prone to self-stigmatization if they had more psychopathological comorbidities. Based on our findings, we aimed to inform the development of adapted stigma reduction efforts and of urgently needed multidisciplinary (legal, psychosocial) support options for refugees in Germany and other host countries (4).

## Materials and Methods

### Recruitment

Participants of the *Sanadak* trial were recruited in Leipzig, Dresden and Halle, Germany, following a multimodal approach (e.g., through multipliers working with Syrian refugees or personal contacts of study nurses) between October 2018 and December 2019. A detailed description of recruitment procedures is given elsewhere (27). Potential participants were invited to a screening interview assessing eligibility criteria as follows: (I) Syrian refugees residing in Germany, (II) 18–65 years old, (III) experienced at least one traumatic event and reported subsequent mild to moderate posttraumatic stress symptom severity (Posttraumatic Diagnostic Scale for DSM-5, PDS-5 total score = 11–59) (28) and (IV) owning a device compatible with the app (Android/iOS). Exclusion criteria were (I) posttraumatic stress symptom severity beyond the range stated above, (II) severe depressive symptoms (Patient Health Questionnaire, PHQ-9 total score ≥ 20) (29), (III) acute suicidal tendencies (Depressive Symptom Inventory-Suicidality Subscale, DSI-SS total score ≥ 3) (30), (IV) current psychotherapy/psychiatric treatment and/or psychotropic medication, as well as (V) females being pregnant. If study eligibility was not fulfilled, individuals were provided with psychoeducation material on mental health care and contact information of local initiatives offering face-to-face support. Five individuals scored slightly above the cutoff for suicidality on the DSI-SS. However, they were included in the study based on their overall clinical impression and ensuring no intention to act. This decision was reached in consensus conferences including study nurses and study psychologists.

### Measures

A detailed overview of the measures implemented in the *Sanadak* trial and the procedure of translation to Arabic (if measures were not available in Arabic) is provided in the study protocol (26). All assessments were in Arabic and took place in the form of structured face-to-face interviews with trained study nurses who were native speakers. The screening assessment included, among others, questions on sociodemographic data and further eligibility criteria outlined above. Upon trial inclusion, participants completed a comprehensive baseline assessment, covering measures of self-stigma and psychopathological symptoms.

*Mental health self-stigma* was assessed using the Self-Stigma of Mental Illness Scale – Short Form (SSMIS-SF) (12). The SSMIS-SF comprises 20 items rated on a 9-point scale ranging from 1 (*strongly disagree*) to 9 (*strongly agree*). There are four subscales, each consisting of four 5-items, which represent consecutive stages in the stigma internalization process: stereotype awareness (*Aware*), stereotype agreement (*Agree*), application to self (*Apply*), and harm to self-esteem (*Harm*). Examples of items are “I think the public believes … most people with mental illness are to blame for their problems” (subscale *Aware*), “I think … most people with a mental illness cannot take care of themselves” (subscale *Agree*), “Because I have a mental illness … I am dangerous” (subscale *Apply*), or “I currently respect myself less because … I am unpredictable” (subscale *Harm*). Sum scores were calculated for each of the subscales, with a possible range of 5 (low level of self-stigma) to 45 (high level of self-stigma). Good reliability and validity have been demonstrated for the SSMIS-SF (12).

*Psychopathological comorbidities* (depression, generalized anxiety, somatization) were determined according to scores of the Patient Health Questionnaire – Depression Module (PHQ-9) (29), the Generalized Anxiety Disorder Scale (GAD-7) (31) and the Patient Health Questionnaire – Somatic Symptom Module (PHQ-15) (32). We used a cutoff of ≥ 10 to indicate clinically significant symptom severity for all three measures (29, 31, 32).

### Statistical Analyses

Statistical analyses were performed in R, version 3.6.3 (33) with RStudio, version 1.3.1056 (34). Descriptive statistics were expressed as absolute numbers, percentages or means with standard deviations. Correlations between self-stigma subscales were calculated as Pearson's *r* bivariate correlation coefficients using pairwise complete observations. Differences between the four self-stigma subscales were tested using one-way repeated measures analysis of variance (ANOVA), which is in line with previous investigations of the progressive model of self-stigmatization based on cross-sectional data (12). In order to assess self-stigma differences between sociodemographic and psychopathological groups, we used independent samples *t*-tests or, in the case of more than two categories, one-way ANOVA. We performed multiple linear regression analyses to test whether a higher number of comorbidities predicted self-stigma scores. We applied a cutoff of ≥2 to indicate higher comorbidity, given that previous research yielded the strongest associations with mental health stigma for multiple comorbidities (35). Regression models were adjusted for age, gender and school-based education. Distribution assumptions for linear models were not violated. All analyses were based on a significance level of α = 0.05.

## Results

### Sample Description

Table 1 shows sociodemographic characteristics of the analysis sample (*N* = 133). The majority of participants were male, single, had a high degree of school education and not more than one psychopathological comorbidity. The age of participants in the analysis sample ranged from 18 to 64 years. Mean posttraumatic stress symptom level (PDS-5) according to the screening assessment was 24.4 (*SD* = 11.1).

**Table 1:** Sociodemographic characteristics of the analysis sample (*N* = 133).

**Variable**	
**Age** (years): *M (SD)*	33.3 (11.2)
**Gender**
Female	51 (38.3)
Male	82 (61.6)
**Education (school-based)**^a^
<12 years	37 (28.2)
≥12 years	94 (71.8)
**Family status**^b^
Single	69 (53.1)
Married	51 (39.2)
Divorced/Widowed	10 (7.7)
**Number of comorbidities**
≤ 1	75 (56.4)
≥2	58 (43.6)

*Entries are n (%) unless indicated differently. M, mean; SD, standard deviation*.

a *Missing data: n = 2, not included*.

b *Missing data: n = 3, not included*.

### Mental Health Self-Stigma Scores

Table 2 shows the mental health self-stigma scores in the analysis sample. One-way repeated measures ANOVA for stigma subscales was statistically significant [*F*_(2.5, 330.6)_ = 73.526, *p* < 0.001, $\eta_{\text{p}}^{2}$ = 0.361]. *Post-hoc* tests with Bonferroni-corrected *p*-values yielded significantly higher scores on *Stereotype awareness* than the other three subscales (*p* < 0.001), and significant differences between higher *Stereotype agreement* and lower *Application to self* (*p* = 0.007). Correlations between self-stigma subscales are shown in Table 3. While scores were not lower for each subsequent stage of self-stigmatization, correlations were higher between more proximate vs. more distal stages of self-stigmatization.

**Table 2:** Mental health self-stigma scores in the analysis sample (*N* = 133).

**Variable**	**Range**	***M (SD)***
**Self-stigma (SSMIS-SF)**
Stereotype awareness^a^	11–45	28.32 (7.48)
Stereotype agreement^b^	5–38	18.82 (6.73)
Application to self^a^	5–39	16.52 (6.60)
Harm to self-esteem^a^	5–43	18.58 (9.52)

*Possible range for SSMIS-SF subscales: 5–45; higher values indicate higher self-stigma; M, mean; SD, standard deviation.*

a *Missing data: n = 1, not included*.

b *Missing data: n = 2, not included*.

**Table 3:** Pearson's r bivariate correlation matrix for SSMIS-SF subscales.

**Variable**	**1**.	**2**.	**3**.	**4**.
1. Stereotype awareness	1.00			
2. Stereotype agreement	0.18*	1.00		
3. Application to self	0.09	0.33***	1.00	
4. Harm to self-esteem	0.01	0.13	0.38***	1.00

*p <0.05, ***p <0.001.

### Associations of Sociodemographic Characteristics and Mental Health Self-Stigma

Univariate comparisons of mental health self-stigma scores across age groups, gender, education and family status are displayed in Table 4. Testing for differences between younger (18–39 years) and older (40–64 years) age groups yielded significantly lower *Application to self* scores for older vs. younger individuals (Cohen's *d* = 0.53). Testing for gender and education differences on self-stigma yielded no significant results. *Post-hoc* tests with Bonferroni-adjusted *p*-values yielded significantly higher *Application to self* scores for single vs. married participants (*p* = 0.003).

**Table 4:** Means and standard deviations of mental health self-stigma scores according to sociodemographic characteristics.

	**SSMIS-SF subscales**			
**Variable**	**Stereotype awareness**	**Stereotype agreement**	**Application to self**	**Harm to self-esteem**
**Age groups**				
18–39 years (*n* = 99)	28.64 (7.28)	18.60 (6.53)^a^	17.37 (6.61)	19.16 (9.40)
40–64 years (*n* = 34)	27.36 (8.10)^a^	19.45 (7.36)^a^	13.94 (5.95)^a^	16.85 (9.70)^a^
*t*-test statistics	*t*_(130)_ = 0.85, *p* = 0.399	*t*_(129)_ = −0.63, *p* = 0.531	*t*_(130)_ = 2.65, *p* = 0.009**	*t*_(130)_ = 1.21, *p* = 0.228
**Gender**				
Female (*n* = 51)	28.86 (7.60)^a^	18.60 (6.95)^a^	16.24 (6.20)^a^	19.44 (8.64)^a^
Male (*n* = 82)	27.99 (7.44)	18.95 (6.63)^a^	16.68 (6.86)	18.06 (10.03)
*t*-test statistics	*t*_(130)_ = −0.65, *p* = 0.518	*t*_(129)_ = 0.29, *p* = 0.773	*t*_(130)_ = 0.37, *p* = 0.710	*t*_(130)_ = −0.81, *p* = 0.422
**Education (school-based)**^b^				
<12 years (*n* = 37)	27.75 (7.41)^a^	19.60 (6.54)^b^	16.72 (6.31)^a^	17.11 (8.55)^a^
≥12 years (*n* = 94)	28.41 (7.57)	18.47 (6.86)	16.47 (6.79)	19.00 (9.90)
*t*-test statistics	*t*_(128)_ = −0.45, *p* = 0.653	*t*_(127)_ = 0.84, *p* = 0.400	*t*_(128)_ = 0.19, *p* = 0.846	*t*_(128)_ = −1.01, *p* = 0.315
**Family status**^c^				
Single (*n* = 69)	29.20 (6.91)^a^	19.78 (6.28)	18.17 (7.03)	19.20 (10.21)
Married (*n* = 51)	27.68 (7.68)^a^	17.66 (7.20)^a^	14.16 (5.28)^a^	17.38 (8.51)^a^
Divorced/Widowed (*n* = 10)	26.40 (9.64)	18.30 (7.90)	15.90 (7.26)	19.70 (10.35)
One-way ANOVA statistics	*F*_(2, 126)_ = 1.00, *p* = 0.372	*F*_(2, 125)_ = 1.44, *p* = 0.241	*F*_(2, 126)_ = 5.7, *p* = 0.004**	*F*_(2, 126)_ = 0.60, *p* = 0.548

Possible range for SSMIS-SF subscales: 5–45; higher values indicate higher self-stigma. **p <0.01, ***p <0.001.

a *Missing data: n = 1, not included*.

b *Missing data: n = 2, not included*.

c *Missing data: n = 3, not included*.

### Associations of Psychopathological Comorbidities and Mental Health Self-Stigma

**Figure 2:**
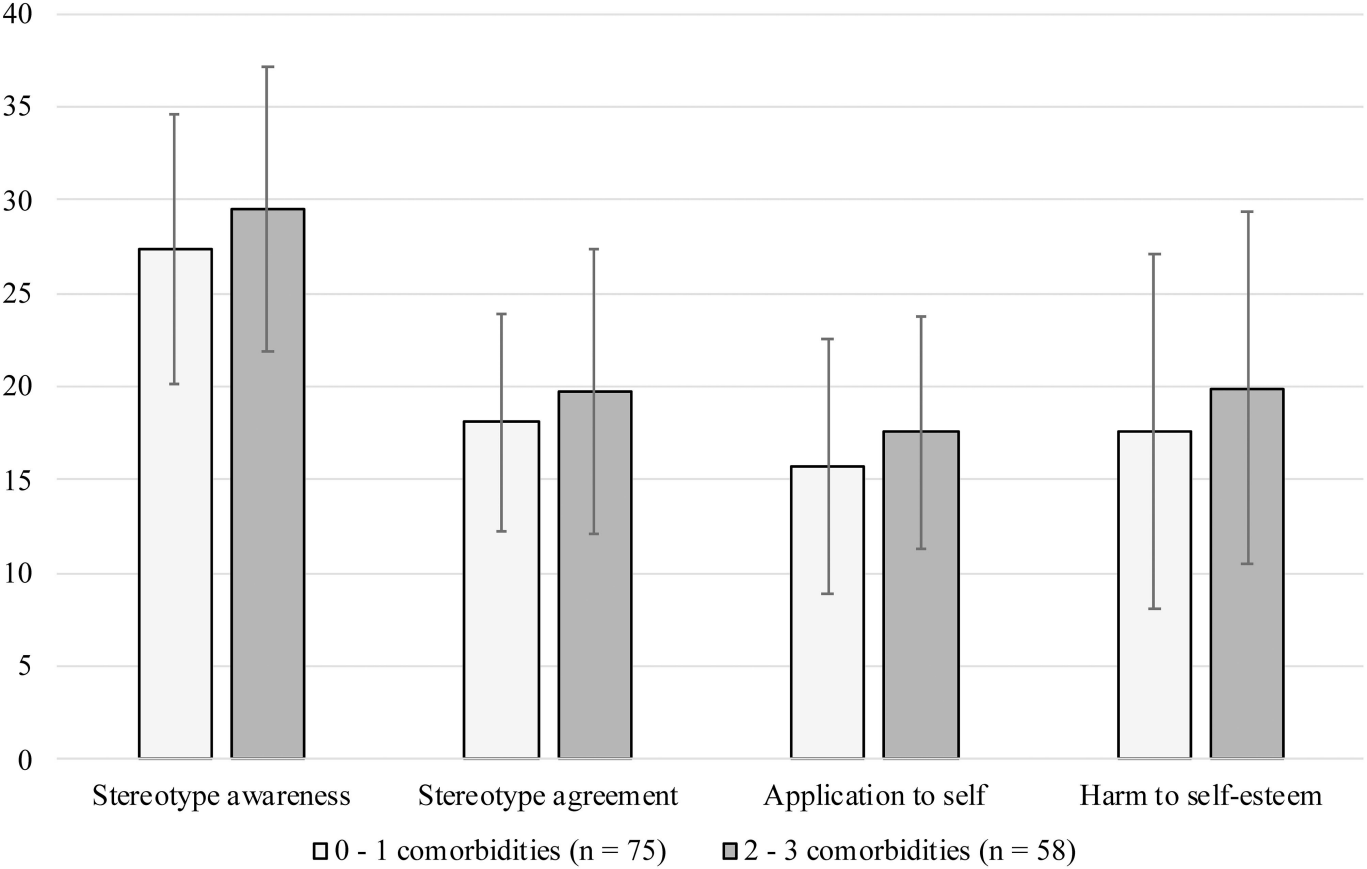
Mental health self-stigma scores (means and standard deviations) according to number of comorbidities.

Self-stigma subscales between individuals with different numbers of comorbidities (see Figure 2) were not statistically significantly different. Multivariate linear regression analyses with mental health self-stigma scores as outcomes (see Table 5) yielded no significant associations of number of comorbidities with *Stereotype awareness* and *Harm to self-esteem*. Being married (in reference to being single) was significantly associated with lower scores on *Stereotype agreement*. In addition, higher age was significantly associated with lower scores on *Application to self* , whereas having ≥2 psychopathological comorbidities (in reference to ≤ 1 comorbidity) was significantly associated with higher scores on that subscale.

**Table 5:** Results of multiple linear regression analyses for predicting mental health self-stigma.

**Variable**	***b***	**95% CI**	**β**	***p***
**Stereotype awareness (*****n*** **=** **127):** ***R***^**2**^ **=** **0.051, adjusted** ***R***^**2**^ **=** **0.003**, ***F***_**(6, 120)**_ **=** **1.07**, ***p*** **=** **0.382**
Age (years)	−0.027	(−0.171; 0.116)	−0.041	0.706
Female gender^a^	1.304	(−1.580; 4.189)	0.085	0.372
Education ≥ 12 years^b^	1.015	(−1.983; 4.013)	0.062	0.504
Family status^c^				
Married	−1.702	(−5.113; 1.709)	−0.111	0.325
Divorced/Widowed	−3.030	(−8.647; 2.587)	−0.110	0.288
≥2 comorbidities^d^	2.224	(−0.490; 4.938)	0.149	0.107
Constant	27.878	(22.894; 32.862)	–	<0.001***
**Stereotype agreement (*****n*** **=** **126):** ***R***^**2**^ **=** **0.071, adjusted** ***R***^**2**^ **=** **0.024**, ***F***_**(6, 119)**_ **=** **1.51**, ***p*** **=** **0.181**
Age (years)	0.109	(−0.021; 0.240)	0.180	0.100
Female gender^a^	−0.042	(−2.663; 2.580)	−0.003	0.975
Education ≥ 12 years^b^	−1.457	(−4.224; 1.309)	−0.096	0.299
Family status^c^				
Married	−3.877	(−6.989; −0.765)	−0.276	0.015*
Divorced/Widowed	−3.383	(−8.489; 1.723)	−0.134	0.192
≥ 2 comorbidities^d^	1.399	(−1.078; 3.876)	0.102	0.266
Constant	17.314	(12.754; 21.875)	–	<0.001***
**Application to self (*****n*** **=** **127):** ***R***^**2**^ **=** **0.164, adjusted** ***R***^**2**^ **=** **0.122**, ***F***_**(6, 120)**_ **=** **3.92**, ***p*** **=** **0.001**
Age (years)	−0.164	(−0.285; −0.042)	−0.274	0.009**
Female gender^a^	0.568	(−1.862; 2.999)	0.041	0.644
Education ≥ 12 years^b^	−0.415	(−2.942; 2.111)	−0.028	0.745
Family status^c^				
Married	−2.332	(−5.206; 0.542)	−0.169	0.111
Divorced/Widowed	−0.181	(−4.914; 4.552)	−0.007	0.940
≥ 2 comorbidities^d^	2.347	(0.061; 4.634)	0.175	0.044*
Constant	21.848	(17.648; 26.048)	–	<0.001***
**Harm to self-esteem (*****n*** **=** **127):** ***R**^**2**^* **=** **0.064, adjusted** ***R**^**2**^* **=** **0.018**, ***F***_**(6, 120)**_ **=** **1.38**, ***p*** **=** **0.229**
Age (years)	−0.146	(−0.329; 0.038)	−0.171	0.118
Female gender^a^	1.791	(−1.892; 5.474)	0.091	0.338
Education ≥ 12 years^b^	2.338	(−1.491; 6.166)	0.110	0.229
Family status^c^				
Married	−0.573	(−4.929; 3.782)	−0.029	0.795
Divorced/Widowed	1.958	(−5.214; 9.130)	0.055	0.590
≥2 comorbidities^d^	2.776	(−0.689; 6.241)	0.144	0.115
Constant	19.744	(13.381; 26.108)	–	<0.001***

b = unstandardized regression coefficient. CI = confidence interval for unstandardized regression coefficients. β = standardized regression coefficient. *p <0.05, **p <0.01, ***p <0.001.

a *Reference category for gender: male*.

b *Reference category for education: <12 years*.

c *Reference category for family status: single*.

d *Reference category for comorbidities: ≤ 1 comorbidity*.

## Discussion

The study aim was to investigate mental health self-stigma and to investigate its associations with sociodemographic and psychopathological characteristics in a sample of adult Syrian refugees with mild to moderate posttraumatic stress symptoms residing in Germany. Forty-four percent of participants in our sample had multiple psychopathological comorbidities. Our results showed higher scores on *Application to self* for individuals who were younger and single, respectively. Moreover, regression analyses yielded significant associations between having multiple comorbidities and higher *Application to self* , controlling for sociodemographic covariates (age, gender, education).

### Mental Health Self-Stigma in the Sanadak Sample

The scores on the four subscales of mental health self-stigma in the current sample broadly aligned with results from clinical non-refugee samples based on the same measure (12, 16, 36). Due to differences in study samples, however, the comparability to our findings is limited. To our knowledge, our work is the first to apply the SSMIS-SF in a refugee sample and further evidence is needed in order to draw conclusions on the severity of mental health self-stigma in different refugee populations. Using the *Brief Version of the Internalized Stigma of Mental Illness Scale* (ISMI-10) (37), a recent study reported mild mental health self-stigma in a sample of Arabic-speaking (predominantly Syrian) refugee outpatients with depressive symptoms in Germany (38). The authors argued that these findings might reflect sampling bias, since participants had already taken the step to seek mental health treatment. These limitations also apply to our sample, considering that the *Sanadak* self-help app is a form of mental health treatment. Thus, our findings might underestimate the true extent of mental health self-stigma among Syrian refugees in Germany. In particular, the level of self-stigma may be higher among those who avoid treatment. Evidence from representative samples is needed in order to draw firm conclusions on this issue. It should also be noted here that the SSMIS-SF items for perceived public stigma (*Stereotype awareness*) might be more difficult to interpret in the case of refugees who have left the societal context that they grew up in and have entered a different cultural and societal sphere in the host country.

Compared to *Stereotype awareness*, our results showed significantly lower scores for the three subsequent stages (*Stereotype agreement, Application to self* , *Harm to self-esteem*) in the self-stigmatization process (see Figure 1). Another hypothesized contrast occurred between *Stereotype agreement* (higher) and *Application to self* (lower). However, scores for *Harm to self-esteem* were not significantly different compared to the preceding two stages. Similar to our findings, previous examinations of the model have yielded inconsistent patterns. For example, Corrigan et al. (12) reported the contrast between the first and the subsequent three stages as the only one consistent difference across three previous studies. A more recent study found differences between, but not within, the first two and the last two stages (13). Other results fully confirmed the assumed trickle down pattern, with significantly lower scores for each subsequent stage in a sample of patients with alcohol dependence (15). Yet, it should be noted that this latter study did not use the 20-item SSMIS-SF, but an adapted version of the 36-item SSMIS (11). In line with the second model assumption and previous findings (12, 13, 15), correlations between more proximate vs. more distal stages of self-stigmatization were higher in the present study. Correlations were strongest between adjacent stages (*Stereotype awareness – Stereotype agreement; Stereotype agreement – Application to self; Application to self – Harm to self-*esteem) and weakest between the most distant stages (*Stereotype awareness – Harm to self-esteem*).

Similar to existing studies, our findings indicated a differentiation between higher perceived public stigma and lower self-stigma, as well as higher correlations between more proximal stages of self-stigmatization. In light of inconclusive evidence, the assumption of a trickle-down process with decreasing self-stigma along the postulated four stages requires further empirical testing and corresponding refinement. The underlying idea of stigma internalization as a progression from public stigma to self-stigma is, however, substantiated by longitudinal empirical evidence (39, 40). Notably, these longitudinal studies conceptualized mental health self-stigma in relation to help seeking. The large variety of mental health stigma concepts and measures in a growing number of studies has been criticized as a hindering factor for consistent stigma research (41). As a response to this concern, a common framework for mental illness stigma conceptualization and assessment has been proposed, integrating the perspectives of the stigmatizers and the stigmatized, and considering different stigma facets in this context (41). We suggest to draw upon this framework in future studies in order to increase the consistency and comparability of empirical evidence.

### Sociodemographic Correlates of Mental Health Self-Stigma

Our results showed that being married (vs. single) was associated with less *Stereotype agreement*, when adjusting for the other sociodemographics and the number of comorbidities. Given the lack of reported sociodemographic characteristics of mental health self-stigma in refugees from Syria, we draw upon a systematic review on mental health stigma in individuals in different Arabic cultural contexts (42). This study reported findings on perceptions of mental illness as shameful and incompatible with marriage. Similarly, a study on mental health stigma in different Muslim communities [the majority of Syrian refugees in Germany are Muslims (3)] reported detrimental effects on marriage prospects (43). This could provide an explanation for the higher levels of mental health self-stigma among single vs. married individuals in our sample.

Results of our multivariate regression model also showed a significantly higher level of stigmatizing views on oneself (*Application to self* ) for younger vs. older individuals. This is in line with results from a Canadian population-based survey which reported significantly lower self-stigma of depression for older adults (44). While a systematic review and meta-analysis found no overall associations between mental health self-stigma and age, the majority (64%) of those studies reporting significant findings indicated a negative relationship (17).

Associations of self-stigma with gender and school-based education were inconsistent and overall not significant. Given that correlates may vary depending on cultural contexts (45), future studies should consider the interplay between sociodemographic factors such as age, gender, religion and family status when investigating mental health self-stigma in Syrian refugee samples.

### Comorbidities as a Risk Factor for Mental Health Self-Stigma

Our data indicated a substantial psychopathological symptom burden among study participants. More than 60% of the *Sanadak* sample scored above the cutoff for at least one comorbid mental disorder. Similarly, results from a recent follow-up examination yielded persistently elevated rates of mental disorders among Syrian refugees in Germany (46). Moreover, a systematic review and meta-analysis reported elevated levels of anxiety, depression, and posttraumatic stress disorder in refugees resettling in high-income countries (47).

Controlling for sociodemographic covariates, a higher number of psychopathological comorbidities was significantly associated with a stronger application of stigmatizing views to oneself (*Application to self* ). We found no such associations for the remaining three self-stigma subscales (*Stereotype awareness, Stereotype agreement, Harm to self-esteem*). Stronger self-stigma for higher symptom severity has been reported before, both in Western samples and in Arabic refugee samples (17, 48, 49). Yet, the question of causality in this context is still unclear, with evidence for both higher symptom burden as a result of a higher self-stigma and vice versa (17). Previous studies have largely focused on self-stigma as a relevant contributor to, rather than consequence of, mental distress. This line of research has shown self-stigma to longitudinally predict negative outcomes such as impaired recovery (50), and worse social functioning (51).

On the other hand, possible reciprocal relationships between self-stigma and symptom severity have been addressed in previous studies (52). In the context of therapeutic intervention efforts, interrelations between symptom change and self-stigma change raise the question of relevant intervention targets and mechanisms (53). Thus, more longitudinal research is needed to further elucidate this chicken-egg problem of mental ill-health and self-stigmatization. In this context, our results underline the relevance of the *Application to self* dimension when working with the progressive model of self-stigma. Considering that Syrian refugees in Germany may be faced to different forms of public stigma and discrimination at the same time, it would be interesting to investigate internalized negative views in this population group following an intersectional approach, i.e., concurrently considering multiple personal characteristics that could be associated with specific discrimination experiences (19, 54). In this way, future studies could expand on existing findings of perceived discrimination predicting depressive and generalized anxiety symptoms in Syrian refugees in Germany (46).

### Limitations

The present study was based on a convenience sample and as such, not representative of Syrian refugees with posttraumatic stress symptoms living in Germany. A second limitation is that our analyses were explorative and based on cross-sectional data, allowing no conclusions on causal associations. Future research may elaborate our findings by investigating trajectories in the self-stigmatization process across time, applying a process-based longitudinal research design. Thirdly, we determined self-stigma and the mental health status of participants using self-report measures so that we cannot rule out biased answers due to social desirability. Fourthly, the comparability of our findings to previous findings is limited by differences in study samples and measures of mental health self-stigma. Fifthly, we did not differentially investigate possible associations between specific psychopathological syndromes and mental health self-stigma. For example, depressive symptoms such as a negative self-image and associated cognitive biases might be closely linked to self-stigmatization. These potential associations should be addressed in future studies. Sixthly, we did not examine possible differences in mental health self-stigma according to participants' asylum status, whereas this aspect might have an effect not only on mental health self-stigma, but on mental health as a whole. Expanding the scope of post-migration stressors in mental health self-stigma research in refugee populations may provide important insights on risk and protective factors on a structural level.

### Implications for Future Research

Previous research has suggested mental health self-stigma as an important barrier to accessing mental health care among refugees (7). Therefore, it is important to address mental health self-stigma in these populations. However, studies in this regard are still scarce and future research is needed to better understand self-stigma in refugees. Such an understanding is an important prerequisite for identifying the specific needs of refugees in order to develop tailored psychosocial intervention approaches. Reducing self-stigma in refugee groups with typically high prevalence of mental disorders is important to facilitate mental health care utilization.

### Conclusion

We provide empirical evidence on mental health self-stigma and its correlates in Syrian refugees with posttraumatic stress residing in Germany, a population in which self-stigma remains not well-studied to date. Younger, unmarried refugees having multiple psychopathological comorbidities appear to be at increased risk for mental health self-stigma, which in turn could be an obstacle to access mental health care. Legal authorities and providers of psychosocial support need to be sensitized to this particular risk association in order to reduce the long-term risk of adverse psychological consequences and to promote social integration. Correlates of mental health self-stigma should be considered in the development of stigma reduction campaigns, as well as efforts to increase access to mental healthcare among Syrian refugees with mental health concerns in Germany.

## Data Availability Statement

The datasets presented in this article are not readily available because of patient confidentiality and participant privacy. Requests to access the datasets should be directed to Alexander Pabst, Alexander.Pabst@medizin.uni-leipzig.de.

## Ethics Statement

The studies involving human participants were reviewed and approved by ethics committee of the Medical Faculty of the University of Leipzig, Germany (ID: 111–17-ek) and was conducted in accordance with the Declaration of Helsinki (World Medical Association 2009) and the ICH guidelines for Good Clinical Practice (GCP). The patients/participants provided their written informed consent to participate in this study.

## Author Contributions

JB contributed to acquisition of the data, formulated the research question, wrote the statistical analysis plan, conducted the statistical analyses, interpreted the data, wrote the manuscript, and gave final approval of the version to be published. AP was the trial statistician, contributed to acquisition of the data, supported in analysis of the data, interpreting the results, and drafting the manuscript, and gave final approval of the version to be published. SR substantially contributed to acquisition of the data, supported in drafting the manuscript, and gave final approval of the version to be published. ML, AR, MN, JD, TG, AK, and H-HK contributed to acquisition of the data, revised the manuscript critically for important intellectual content, and gave final approval of the version to be published. SR-H conceptualized and designed the study, supported in interpreting the data, revised the manuscript critically for important intellectual content, and gave final approval of the version to be published. All authors contributed to the article and approved the submitted version.

## Conflict of Interest

The authors declare that the research was conducted in the absence of any commercial or financial relationships that could be construed as a potential conflict of interest.
